# Blunt Traumatic Innominate Artery Pseudoaneurysm Endografting without Heparin Due to Severe Brain Injury

**DOI:** 10.1055/s-0041-1739486

**Published:** 2022-05-31

**Authors:** Derek P. Nieber, C. Taylor Lewis, Rajeev Dayal, Konstantin Khariton, Samuel J. Lang, Charles A. Mack

**Affiliations:** 1 Department of Cardiothoracic Surgery, Weill Cornell Medicine, New York, New York; 2 Department of Surgery, Weill Cornell Medicine, Flushing, New York

**Keywords:** innominate artery pseudoaneurysm, blunt trauma, neurological injury

## Abstract

Blunt traumatic innominate artery injuries occur in polytrauma victims who have suffered high-speed motor vehicle collisions. Their associated injuries may preclude the use of heparin and affect surgical management and perioperative neurological risk. The uniqueness of this case is combining the arterial injury repair with a severe progressive neurological injury that prohibited standard perioperative antiplatelet or anticoagulent use.

## Introduction

Blunt traumatic innominate artery (IA) injuries occur in patients who have endured high-energy mechanisms of injury and are a leading cause of mortality. 1 IA injuries are suspected from a seat belt sign across the neck and chest, neck hematoma, hemodynamic instability, pulse asymmetry, and widened mediastinum on radiography. 2 3 Diagnosis is made with computed tomography (CT) and supplemented with angiography. Management is surgical, by open, endovascular, and hybrid techniques. 3 4 Hemodynamically unstable patients who require emergent repair may have associated neurological injuries that preclude the use of heparin, thereby requiring alternative surgical approaches at elevated perioperative stroke risk. No comparative data exist to guide decision-making.

## Case Presentation

**Fig. 1:**
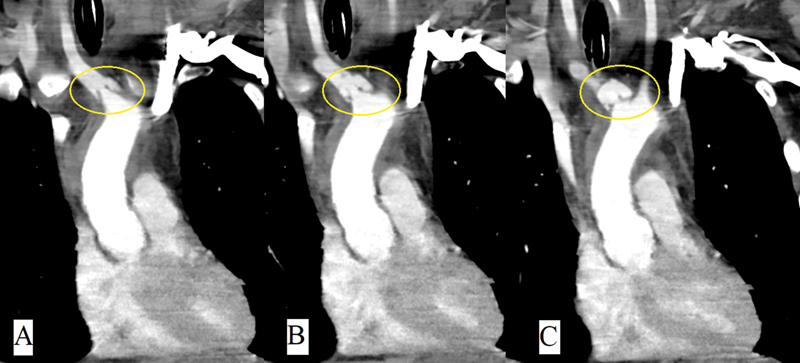
( **A** – **C** ) Coronal views of computed tomography of the chest with intravenous contrast demonstrating proximal innominate artery pseudoaneurysm. Yellow circle identifies injury.

A 27-year-old healthy male presented as an unhelmeted motorcyclist hit by a car. He arrived obtunded with a heart rate of 163, blood pressure 213/126 mm Hg, and neck swelling. He was intubated for a Glasgow Coma Scale score of 3. Focused assessment with sonography was negative. Chest radiograph demonstrated widened mediastinum. CT imaging revealed diffuse axonal injury, subdural hematoma, subarachnoid hemorrhage, and pseudoaneurysm of the proximal IA ( Fig. 1 ). Additional injuries included: femoral, mandibular, cervical spinous process, and rib fractures. Initial coagulation studies demonstrated mild coagulopathy, activated partial thromboplastin time: 37.6 seconds, prothrombin time: 14.9 seconds, international normalized ratio: 1.29, and platelet count: 123 × 10 ^9^ /L.

His Injury Severity Score was 43. His Trauma and Injury Severity Score predicted a 32.5% probability of survival.

The patient returned to the trauma bay for ventriculostomy placement 2.5 hours after arrival. Repeat CT demonstrated a new 4-cm frontal lobe intracerebral hemorrhage (ICH), 2-mm midline cerebral shift, and progressive mediastinal hematoma. Emergent IA repair was recommended after multidisciplinary assessment. His progressive ICH precluded anticoagulation use. Endovascular IA repair with possible sternotomy and off-pump IA bypass was undertaken without anticoagulation 4.5 hours after arrival.

The patient was positioned supine and prepared from chin to knees. A left common femoral artery (CFA) 5-French (Fr) sheath and a right CFA 8-Fr sheath with a Perclose Proglide (Abbott Vascular Inc., Santa Clara, CA) were inserted percutaneously. An aortogram confirmed an IA pseudoaneurysm 1 cm from its origin. Through wire access was obtained to accommodate a larger diameter stent graft if required, so the right common carotid artery (CCA) was exposed surgically using an incision medial to the sternocleidomastoid muscle. A 6-Fr sheath was placed into the right CCA.

**Fig. 2:**
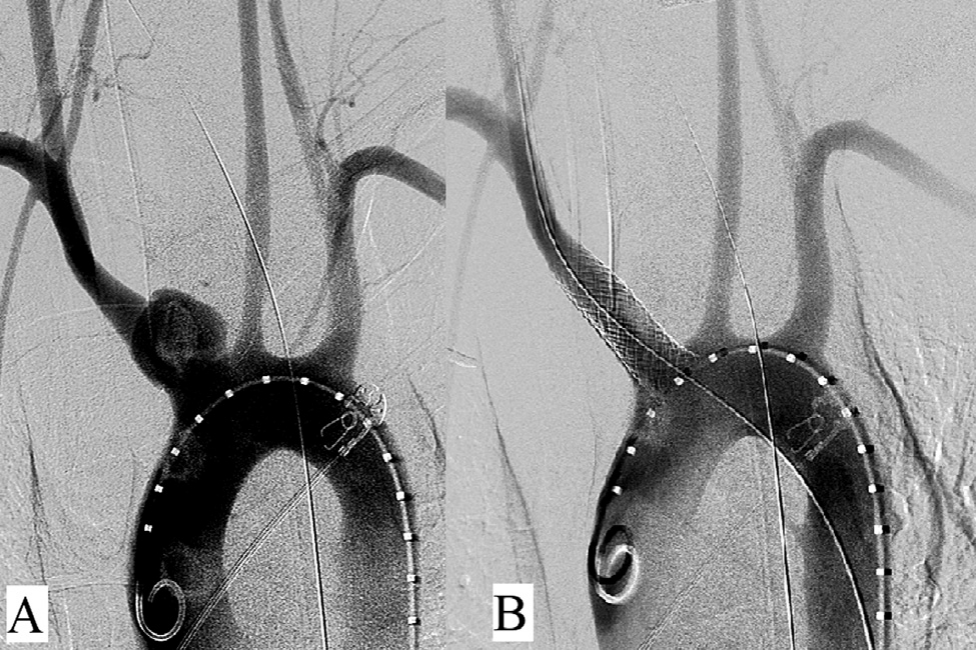
Intraoperative aortograms. ( **A** ) Initial diagnostic image. ( **B** ) Completion with overlapping endografts without pseudoaneurysm filling (right).

A 0.035 Terumo Glidewire (Terumo Medical Corporation, Somerset, NJ) was advanced from the right CFA into the aortic arch and snared with an En-Snare (Merit Medical Systems, South Jordan, UT) via the right CCA and externalized ( Fig. 2 ). A 8-Fr by 70-cm Flexor Ansel (Cook Medical, Bloomington, IN) hydrophilic sheath was placed into the aortic arch and two overlapping 11 mm × 39 mm, and 11 mm × 29 mm, balloon-expandable stent grafts (Viabahn VBX, W.L. Gore & Associates, Flagstaff, AZ) were deployed in the IA and dilated using a 16 mm × 40 mm XXL (Boston Scientific Corporation, Marlborough, MA) balloon. Completion angiogram confirmed successful repair. The right CCA sheath was removed and the arteriotomy repaired. The left CFA was closed with a Vascade Closure device (Cardiva Medical, Santa Clara, CA). The patient was transfused intraoperatively with 5-unit red blood cells, 4-unit plasma, and 1-unit platelets. No intravenous heparin was administered.

Postoperatively, he received a hypertonic saline drip and cerebrospinal fluid drainage for intracranial hypertension. CT imaging on hospital days 3 and 17 demonstrated stability of his ICH and no cerebral infarcts. Chemical deep vein thrombosis prophylaxis was started on day 3. The patient underwent repair of his orthopaedic injuries, tracheostomy, and percutaneous gastrostomy. After 7 weeks, he was discharged to a skilled nursing facility speaking, following commands, and working with physical therapy. CT angiography prior to discharge verified successful IA repair.

## Discussion

IA injuries are the second most common blunt traumatic thoracic vascular injuries. 2 They are due to simultaneous neck extension and thoracic compression that results in aortic arch fixation between the manubrium and spine and heart displacement to the left which causes IA injuries near its origin from longitudinal forces. 3 Bovine arch anatomy is thought to predispose to blunt injury. 4 Blunt IA injuries are repaired emergently or delayed to address other injuries if the patient is hemodynamically stable.

Open repair techniques include the following: (1) interposition grafting or end-to-end repair for mid/distal injuries; (2) aortoinnominate bypass using partial aortic cross-clamp; and/or (3) circulatory arrest and partial arch replacement for proximal injuries on cardiopulmonary bypass. 2 3 4 Methods of intraoperative neuroprotection include unilateral perfusion of the CCA, selective shunting if the right CCA stump pressure is <50 mm Hg, and deep hypothermia and circulatory arrest. 2 3 4 5

Endovascular repair techniques range from IA stenting to total aortic arch endografting with great vessel revascularization via fenestration or debranching and extra-anatomic bypass. 1 6 7

Severe neurological injuries can be a contraindication to using intravenous anticoagulation during IA repair. At present, no comparative data exist that quantifies the perioperative risk of stroke from repair of traumatic IA injuries without heparin. A review of 60 patients who underwent open repair for blunt IA injuries by Hirose and Gill reported a 4.5% rate of stroke without comment on perioperative anticoagulation use. 5 Other authors have reported open and endovascular IA and aortic repairs after blunt trauma without anticoagulation and without stroke in limited series and case reports. 1 2 3 4 6

A recent meta-analysis of 1,969 patients who underwent elective isolated and combined IA and CCA interventions reported 30-day stroke rates of 3.8% for isolated open IA/CCA, 2.8% for hybrid endovascular CCA and open internal carotid artery, and 1.1% for isolated IA/CCA endovascular repairs. 7 Modern cerebrovascular interventions use perioperative single- or dual-antiplatelet agents in addition to intraoperative intravenous heparin to target activated clotting time >250 seconds but practices vary. Emergent IA interventions in polytrauma patients with labile hemodynamics and intracranial hypertension are likely to confer increased stroke risk compared with the elective setting but this is unproven.

Our patient's enlarging mediastinal hematoma and hemodynamic instability were felt to require emergent IA repair without heparin in the setting of progressive ICH and unknown neurological examination. The proximity of the injury to the aortic arch initially discouraged an endovascular approach. However the neurological risks of exacerbating intracranial hypertension and interrupting cerebral perfusion during a sternotomy with partial aortic cross clamping and IA bypass warranted an endovascular attempt without heparin.

Externalized through wire access via the right CCA would have allowed us to address a more proximal injury or larger diameter IA using an iliac limb extension graft without advancing devices distally into the right CCA, theoretically reducing the risk of cerebral embolism and increasing technical success rate. Despite carotid access, no evidence of embolic stroke was identified.

Managing blunt IA injuries in a multisystem trauma patient is complex. Our experience hopes to encourage future surgeons to find acceptable surgical interventions for these often young and critically ill patients despite contraindications to heparin and the paucity of data available to guide decision-making or predict perioperative neurological complications.

## References

[1] Abi-Chaker A M Jones K M Sanchez P Sasson J Li X Rey J Successful revascularization of aortic arch in a 39-year-old blunt trauma patient with acute diffuse axonal injury without the use of systemic anticoagulation Ann Vasc Surg 2017 44 4180 4.18E7 10.1016/j.avsg.2017.03.177 28499862

[2] Symbas J D Halkos M E Symbas P N Rupture of the innominate artery from blunt trauma: current options for management J Card Surg 2005 20 05 455 459 16153279 10.1111/j.1540-8191.2005.00111.x

[3] Karmy-Jones R DuBose R King S Traumatic rupture of the innominate artery Eur J Cardiothorac Surg 2003 23 05 782 787 12754033 10.1016/s1010-7940(03)00032-0

[4] Wells P Estrera A Blunt traumatic innominate pseudoaneurysm and left common carotid occlusion with an associated bovine aortic arch J Thorac Cardiovasc Surg 2005 130 03 928 929 16153969 10.1016/j.jtcvs.2005.03.005

[5] Hirose H Gill I S Blunt injury of the innominate artery: a case report and review of literature Ann Thorac Cardiovasc Surg 2004 10 04 218 223 15458372

[6] Volpe P De Caridi G Serra R Alberti A Massara M Successfully kissing stent of innominate artery and left common carotid artery subsequent to blunt injury, in the setting of a bovine aortic arch Ann Vasc Surg 2020 64 4.1E9 4.1E12 10.1016/j.avsg.2019.10.047 31639480

[7] Robertson V Poli F Saratzis A Divall P Naylor A R A systematic review of procedural outcomes in patients with proximal common carotid or innominate artery disease with or without tandem ipsilateral internal carotid artery disease Eur J Vasc Endovasc Surg 2020 60 06 817 827 32928666 10.1016/j.ejvs.2020.06.040

